# Morbilliviruses: Entry, Exit and Everything In-Between

**DOI:** 10.3390/v11111036

**Published:** 2019-11-07

**Authors:** Dalan Bailey

**Affiliations:** The Pirbright Institute, Ash Rd., Guildford GU24 0NF, UK; dalan.bailey@pirbright.ac.uk; Tel.: +44-1483231012

Morbilliviruses are important pathogens, to the point that they have shaped the history of human and animal health. Measles virus (MeV) is well recognised as one of the most significant killers in the history of human viral diseases, while viruses like rinderpest (RPV) have played a critical role in shaping veterinary care, vaccination and eradication strategies. Accordingly, these viruses have been the focus of intense research and various aspects of their virology are well understood and well characterised, for instance how MeV receptor usage determines the tropism and progression of disease. In this Special Issue we gathered a range of primary data and review articles to reflect the diversity of ongoing research on morbilliviruses.

Jo and colleagues investigated the origin of canine distemper virus (CDV) strains responsible for epidemics in seals, identifying a novel clade, with ancestral origins, as the causative strain [1]. This focus on morbillivirus infections in marine mammals was also addressed in two reviews by Kennedy et al., and Ohishi et al., who summarised the mechanistic evidence that is helping researchers to understand the genetic determinants of host range and pathogenesis [2,3]. One of the key factors determining host-range is the capacity of various morbilliviruses to use cognate and non-cognate host SLAM proteins to enter cells. Indeed, Fukuhara et al. identified a number of important host restrictions at this virus-host interface [4]. Combining both mechanistic and epidemiological data, the review by Duque-Valencia et al. on CDV transmission provides further interesting insights into the processes that drive morbillivirus evolution [5]. In related work, Muñoz-Alia et al. compared antibody mediated neutralisation of MeV and CDV to identify factors constraining the evolution of new morbillivirus serotypes [6]. In recent years there has also been much focus on the identification of novel morbilliviruses, in related mammalian hosts. Sieg et al. reported the identification of a new genotype of the recently identified feline morbillivirus, greatly expanding our understanding of the diversity of this virus in nature [7].

From a more basic virology perspective two publications addressed interactions between the viral envelope proteins and the host cell. Tiwarekar et al. identified competitive interactions between the host protein KDELR2, MeV F and H proteins, and molecular chaperones involved in endoplasmic reticulum processing [8]. Separately, research from my lab identified that morbillivirus H proteins are a target for the host-cell interferon stimulated protein BST2/tetherin [9]. 

Looking into the future there is a realistic possibility that other morbilliviruses, besides RPV, may be eradicated. Kreidl et al. discussed methods for identifying susceptible sub-populations during measles vaccination campaigns [10], which may provide a useful tool in countries where vaccination rates are dropping due to misplaced fears about vaccine safety. The other hope for eradication is peste des petits ruminant virus (PPRV), with the OIE and FAO recently launching a global strategy for eradication. To that end, Eloiflin et al. identified a number of mutations within the PPRV live attenuated vaccine which may help to understand the molecular nature of attenuation [11].

Finally, I would like to acknowledge all the authors, editors, and reviewers who helped to make this issue a reality, both at Viruses, and also in the wider academic community.
